# The application of a CART model for forensic human geolocation using stable hydrogen and oxygen isotopes

**DOI:** 10.1038/s41598-022-25394-w

**Published:** 2022-12-07

**Authors:** Momoko Ueda, Lynne S. Bell

**Affiliations:** School of Criminology, Centre for Forensic Research, Simon Fraser University, Burnaby, BC V5A 1S6 Canada

**Keywords:** Biological anthropology, Stable isotope analysis

## Abstract

The utility of stable hydrogen and oxygen isotope analysis of human tissues for geolocation is an important area of study within forensic science. This study aimed to first validate the latitudinal relationship between stable hydrogen and oxygen isotopes in drinking water and human keratinous tissues through the analysis of human samples with known geographical origin. And secondly, to explore the use of classification and regression tree (CART) models to geographically classify individuals based on the stable isotope values of tissues themselves. Human hair and toenails were collected from four distinct study sites across Canada. The comparison of stable isotope values in drinking water and human tissues produced low R^2^ values indicating that linear models may not fully explain the variation observed for both hydrogen and oxygen values. Additionally, large intrapopulation variations were observed for Canadian cities and highlights the importance of understanding the regional isotopic spread of human values. Further, this study demonstrated that a closed group of unknown individuals known to have originated from a limited number of geographically distinct regions may be classified into their respective groups through the use of CART models. The potential for the CART model approach for human geolocation presents a promising new tool.

## Introduction

The analysis of stable hydrogen (*δ*^2^H) and oxygen (*δ*^18^O) isotopic compositions of human tissues is important for identifying unknown human remains as it can provide insight into the possible geographical origins of individuals. Stable hydrogen and oxygen isotopes in human tissues are primarily influenced by direct and indirect water intake, which varies predictably with geography^1,2^. Such geographical variance can provide an opportunity to predict the origins of an individual through the use of linear regression models^3–5^. However, much of the current isotopic work on human tissues has been carried out on archaeological samples^6^ with presumed geographical information. Thus, validation work must be conducted on contemporary human tissue samples with geographically and temporally known data, especially for forensic uses. Furthermore, it has been shown that isotopic variations in recently formed human tissues can exist for a single geographical area. The isotopic spread of *δ*^18^O values of enamel was 4‰ for Metro Vancouver in British Columbia, Canada^4^, and although explained by the large isotopic range of tap water that supplies the regional district^7^, the intrapopulation variation is larger than what has previously been estimated for any geographical region^8,9^. Thus, isotopic values obtained from a single tissue cannot represent the entire geographical area. The aim of this work was therefore to conduct a validation study by analyzing multiple human hair and toenail samples with known geographical information from four constrained study sites across Canada. Furthermore, in hopes to increase the accessibility of the application of stable isotope analysis for human geolocation by non-experts, this study explored the possibility of utilizing the classification and regression tree (CART) model as a predictive tool to help with human geolocation. The output of the CART model is intuitive and can be useful for classifying a closed group of individuals with some knowledge of their possible geographical origins prior to analysis. Additionally, an advantage to this approach is that the models can be developed from the stable isotope delta values of the tissues themselves.

Human hair and nail tissues are commonly analyzed for their isotopic compositions to gain insight into the individuals' lifestyle, dietary choices, residence patterns, and travel history^3,10^. The analysis of keratinous tissues is often preferred over other tissues for forensic studies as they can be obtained non-invasively. Further, hair and nails continue to grow throughout life, are generally resistant to diagenesis, and thus store more recent information. It is also possible to retrieve temporal data from hair as the growth rate of human head hair is well known at approximately 1 cm per month^11^ and where the segment of hair closest to the scalp contains the most recent information compared to the distal ends. Fingernails grow faster than toenails at approximately 3.5 mm per month compared to toenails at an average rate of 1.6 mm per month^12^. The distal ends of nails contain the oldest information. While several studies have investigated the usefulness of analyzing isotopes in fingernails for geolocation^13–17^, very little is known for toenails.

The stable hydrogen and oxygen isotope compositions of keratinous tissues are suggested to be mainly influenced by drinking water^18^. Drinking water is most often derived from local precipitation, and the isotope values vary spatially in a predictable pattern^19^. The stable isotope ratios of hydrogen (^2^H/^1^H) and oxygen (^18^O/^16^O) in meteoric water vary geographically depending on temperature, latitude, elevation and distance from the coast^20^. However, the relationship between hydrogen in hair and water is complex as the hydrogen isotope compositions of hair are more heavily influenced by dietary input than oxygen. Further, the correlation between hydrogen in drinking water and hair is limited to the non-exchangeable hydrogen atoms not bound to oxygen or nitrogen. It is estimated that approximately 9% of hydrogen in human hair is isotopically exchangeable with ambient water and atmosphere^18^. Nonetheless, hydrogen and oxygen in hair and nails have been suggested to be good indicators to geographically place individuals on a spatial map as they closely reflect the isotopic patterns of local precipitation or drinking water.

One of the main approaches for inferring an unknown individual's geographical origin is the development of a spatially interpolated model of the stable isotope compositions of human tissues^21^, otherwise called isoscapes. This is achieved by converting the baseline environmental isotope data into human values using existing linear conversion equations^3,5,16,22,23^. The prediction map for human tissues can then be used to geographically assign unknown individuals based on the consistencies of the isotopic values with the unknown samples. However, such an approach may be limited to large-scale population-level studies as the isotopic gradients are shown at a low geographical resolution and fall short of representing intrapopulation isotopic variations. Variability, as measured by the deviation from the linear correlation line between stable hydrogen and oxygen isotopes in water and human hair, can often be attributed to physiological differences and dietary preferences even amongst individuals from the same geographical locale^3^. Modern studies have observed such variability despite the assumption that the globalization of food systems has led to dietary homogeneity in industrialized countries. Thus, spatial models should be used cautiously, especially with the understanding that large intrapopulation variations can exist. Additionally, utilizing advanced mathematical or statistical models may be less accessible to non-experts where the interpretation of the results can be difficult. A more intuitive approach to human provenancing may be through the development and use of classification models such as the CART model^21^. CART models can be useful for closed populations or where there is a clear understanding of the number of end member groups from which the population originated^24^ and provide an opportunity for individuals to be assigned to their respective groups based on the isotopic values of their tissues. Results produced from CART models have high interpretability and allow non-experts to utilize the model to classify individuals based on the predicted geographical origins.

Thus, this study aimed to validate the understanding of the relationship between stable hydrogen and oxygen isotope compositions in human head hair and toenails with latitude, through the collection of tissues samples with known geographical information. Additionally, this study explored the possibility of using hydrogen and oxygen values in human head hair and toenails to predict an individual's geographical origin from a closed Canadian population using the CART model, which can practically be applied by investigators to assist in missing persons cases.

## Methods

### Ethics permission

Permission to undertake this study was granted by SFU's Office of Research Ethics, on behalf of the Research Ethics Board with the permit issue number [2015s0125]. This research was conducted in accordance with the University Policy R20.01: Ethics Review of Research Involving Human Participants (http://www.sfu.ca/policies/gazette/research/r20-01.html) with informed consent from all subjects.

### Study sites

Four main study sites were selected covering geographically distinct areas across Canada. The sites spanned east (64.3645° W) to west (123.138° W) and north (63.73664° N) to south (44.38931° N). The four study areas were Site1: Vancouver in the province of British Columbia (BC), Site 2: Orillia in the province of Ontario (ON), Site 3: Iqaluit in the Canadian Northern Territory of Nunavut (NU) and Site 4: Wolfville in a Maritime and Atlantic province of Nova Scotia (NS). Smaller communities in and around the designated study sites were also sampled as part of the study sites.

**Figure 1:**
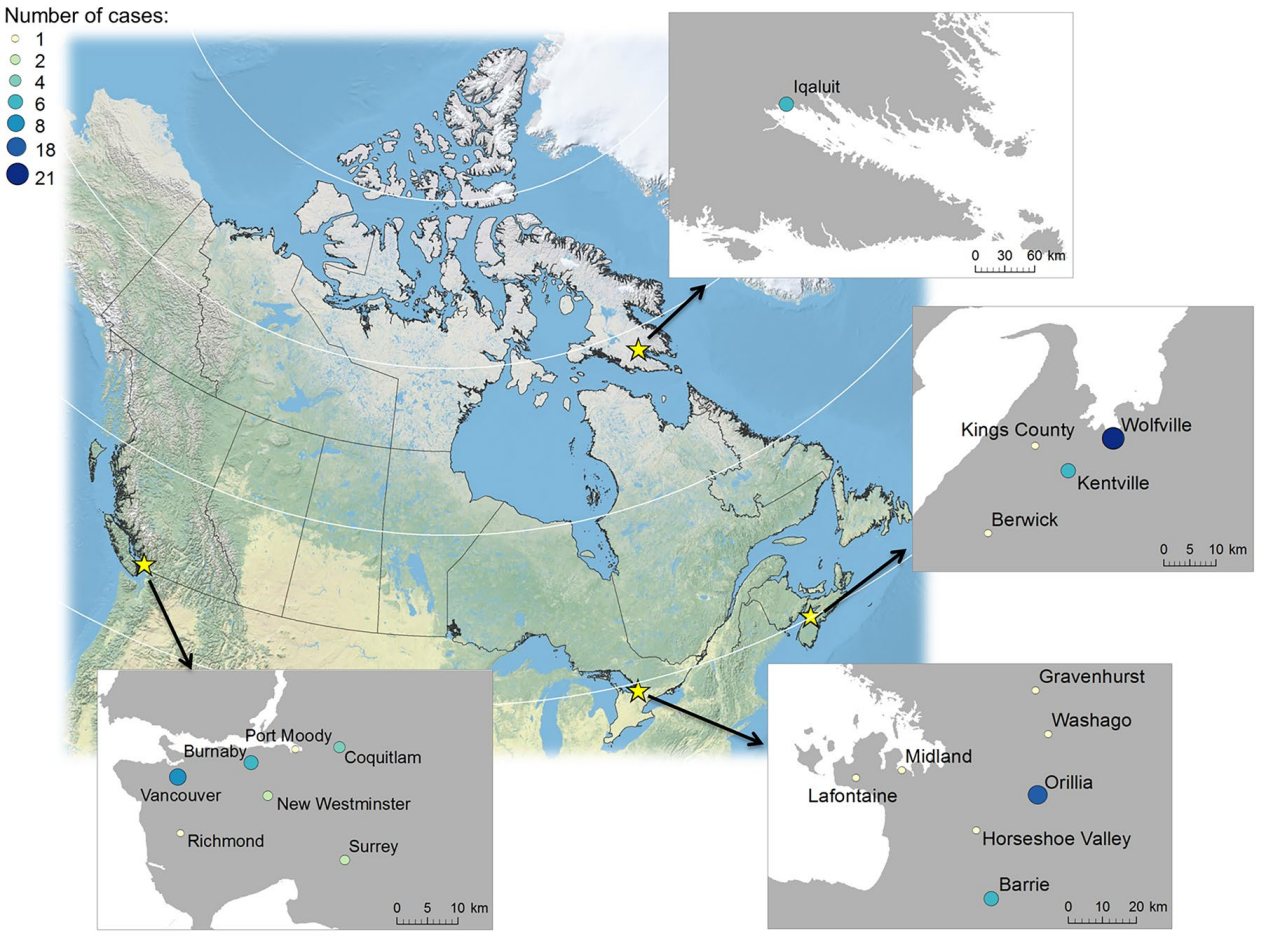
Map of Canada with the four main study sites. Site 1: Vancouver, Site 2: Orillia, Site 3: Iqaluit, Site 4: Wolfville. The map was generated using ArcGIS version 10.8.2 (https://www.esri.ca/en-ca/home).

Site 1, Vancouver (49.26087° N, 123.114° W), is one of the 21 municipalities within the regional district of Metro Vancouver and represents a large conurbation. Metro Vancouver has a population of approximately 2.5 million^25^ and is located in western Canada (Fig. 1). Drinking water for the regional district is overseen by the Metro Vancouver Water District and relies mainly on three main watersheds. The watersheds are located north of the district and supply much of the drinking water for the entire regional district, supplemented by groundwater aquifers in certain areas^26^. Two of the three watersheds, the Capilano and Seymour watersheds, are treated at the Seymour-Capilano Water Filtration Plant, and water from the Coquitlam watershed is treated at the Coquitlam Water Treatment Plant. Treated drinking water is delivered across the regional district through a complex network of interconnected pipes monitored hourly by the Water District of Metro Vancouver. The distribution system and the tap water stable hydrogen and oxygen isotope compositions across the regional district are now well known^7,26^. Site 1 also included samples from Burnaby, Surrey, Port Moody, Coquitlam, New Westminster, and Richmond, all of which are municipalities of Metro Vancouver.

Site 2, Orillia (44.60921° N, 79.4176° W), represents a smaller conurbation with a population of about 30,000^27^. Geographically, it is part of Simcoe County in Ontario. The city is adjacent to the Great Lakes in eastern Canada and mainly draws drinking water from Lake Couchiching^28^. Two groundwater sources also contribute to the drinking water supply for the city. Site 2 sampling sites included nearby cities within the geographical region of Simcoe County, such as Barrie, Horseshoe Valley, Washago, Lafontaine, and the Township of Midland. Gravenhurst, although a part of Muskoka Region, was also included in Site 2 given the proximity to Orillia (Fig. 1). Barrie, Washago, and Gravenhurst rely mainly on drinking water drawn from the surface water bodies of Kempenfelt Bay, Lake Couchiching, and Lake Muskoka, respectively^29–31^. Horseshoe Valley, Lafontaine, and Midland, on the other hand, rely on groundwater sources^32–34^.

Site 3, Iqaluit (63.73664° N, − 68.5227° W), is the capital city of Nunavut and is situated in the most northern area of the four study sites. Iqaluit is a small community with approximately 7000 people, and where over half of the population identifies as Inuit^35^. Lake Geraldine is the primary source of drinking water supplying the city, with Apex River as the secondary source^36,37^. Chlorine-treated water is delivered to residents via a piped system, but many also rely on delivery via tank trucks. Further, untreated raw water sources are also consumed by individuals who prefer to follow traditional cultural practices, similar to other Inuit communities^38^. However, the main drinking water source is a surface water source; thus, the isotopic values of those water should closely reflect local precipitation values.

Finally, Site 4, Wolfville (45.09141° N, 64.3645° W), is located in the easternmost part of Canada of the study sites and is a small rural community of approximately 4000 people. Wolfville is one of the three towns of Kings County in Annapolis Valley of southwestern Nova Scotia, along with the Town of Berwick and the Town of Kentville^39^. The county has an overall population of 60,600 as of 2016^40^ and is the most highly populated area in the province. The county's primary drinking water source is groundwater, with municipal and private wells drawing water from the Cornwallis watershed^41–43^.

### Human tissue sample collection and analysis

Human head hair samples and toenail clippings were collected from living donors with informed consent. 82 hair samples and 39 toenail samples from 86 individuals were collected across Canada's four main study areas (see Supplementary Table S1. online). Of the 82 hair samples, 23 were from Site 1, 25 from Site 2, 6 from Site 3 and 28 from Site 4. For toenails, 13 were from Site 1, 10 from Site 2, 6 from Site 3 and 10 from Site 4. *δ*^2^H and *δ*^18^O values were measured from all hair and toenail samples except for one hair sample (H52), where *δ*^2^H values could not be measured. Collection forms were used to obtain geographical information about the donor's residence, and only those individuals who had resided in the same area for a minimum of 8 consecutive months were included in this study. Individuals were also asked to self-exclude from the study if they had smoked during the past 8-month period, had chemotherapy, or had used nail polish or hair dyes. Each sample was identified with a code to ensure the anonymity of the donors. Cut head hair and toenail samples were stored in borosilicate glass vials until analyses at the Pacific Centre for Isotopic and Geochemical Research. Hair and nail samples were treated before analysis by soaking in chloroform/ethanol mix for 2 h, then rinsed with de-ionized water. They were then placed in open beakers in an evacuated desiccator and allowed to equilibrate with laboratory air. 150–200 µg of hair or nail were placed in a silver capsule and loaded into the zero-blank autosampler. Samples were analyzed using the comparative equilibration method^3,44^ against known human hair and toenail reference samples. Both hair and nail samples were analyzed in continuous flow mode by pyrolysis in a Thermal Combustion Elemental Analyser attached to a Thermo-Finnigan DeltaPlusXL mass spectrometer. Isotopic results are reported in per mil (‰) and calculated using the delta (*δ*) notation following the equation^45^:

$${\delta }^{i}E= \frac{\left(\frac{{}^{i}E}{{}^{j}E}\right)sample- \left(\frac{{}^{i}E}{{}^{j}E}\right)standard }{ \left(\frac{{}^{i}E}{{}^{j}E}\right)standard}$$

The *δ* values refer to the relative differences between the sample and the standard where the ratios of the heavier (*i*) and lighter (*j*) isotopes are measured for a given element (*E*). Three to five analyses were run of each sample with five analyses each of the standards and are averaged for the reported result. The measurement uncertainties as calculated by taking the average of standard deviations was 1.67 for *δ*^2^H in hair, 0.85 for *δ*^2^H in toenails, and 1.96 for *δ*^18^O in hair and 0.89 for *δ*^18^O in toenails. The analyses were corrected for isotopic fractionation and normalized using two international laboratory standards, which are reported cf. international water standards of Vienna Standard Mean Ocean Water (VSMOW) and Vienna Standard Light Antarctic Precipitation (VSLAP). The analytical precision as measured by taking the average standard deviations of *δ*^2^H from the standards are 1.70‰ and 1.39‰ and of *δ*^18^O are 0.67‰ and 0.53‰ for NBS42 and NBS43, respectively, for both hair and toenail samples.

### Tap water data collection

A single tap water sample from Site 4 was collected in Wolfville, NS, during the month of October 2018. Because the same watershed supplies Wolfville as that of Kentville, NS, and Berwick, NS, the isotopic value was also applicable to all three cities in Site 4. Water was filled to the top of an air-tight screw-top borosilicate glass vial and kept in the refrigerator to avoid evaporation. The sample was run through the Los Gatos Triple Liquid Water Isotope Analyzer (TLWIA-45-EP-2013) coupled with CTC Analytics LEAP Technology PAL auto-sampler. Los Gatos Standards 1 to 5A were used as standards with a precision of ± 0.1‰.

For all other study sites, tap water data were collected from existing published sources and searched through available data on the Waterisotope Database^46^. The Waterisotope Database compiles water isotope data from various contributors and has a significant amount of internationally represented water isotope data. It is publicly accessible and is a valuable tool for searching any existing water isotope data, including tap water, surface water, and groundwater. It does not include information on the water source but rather on the geographical location in which the sample was collected. Hence, it was vital for this research to ensure that the data obtained from the database were reflective of the entire city. Data collected from the Waterisotope Database becomes extremely useful for those cities that rely mainly on a single water source. Isotopic data of the water sources were retrieved when no tap water data were available for that city. *δ*^2^H and *δ*^18^O values for annual local precipitation water were also calculated using the Online Isotope Precipitation Calculator^47^ for each study location. *δ*^2^H and *δ*^18^O values for Metro Vancouver cities (Site 1) were taken from Ueda and Bell's^7^ study on Metro Vancouver tap water. *δ*^2^H and *δ*^18^O values for each Metro Vancouver municipality were calculated by determining the fractional contribution of each water source to the main Metro Vancouver municipal water supply as outlined in Ueda and Bell^4^.

### Statistical analysis

A one-way multivariate analysis of variance (MANOVA) test was conducted to determine whether statistically significant differences exist between mean *δ*^2^H and *δ*^18^O values in hair and toenails obtained from the four sampling sites at the α = 0.05 level. The normality of data was tested using the Shapiro–Wilks' method^48^. The Wilcoxin's test^49^ was used for any non-normal datasets. The ordinary least squares method was applied to measure the relationship between stable hydrogen and oxygen isotope compositions of both hair and toenails as well as for drinking water and hair, drinking water and toenails, local precipitation values and hair, and local precipitation values and toenails. R-squared (R^2^) values were calculated as the goodness-of-fit measure^50^. Stable hydrogen and oxygen isotopes in hair and toenails from the same individual were also compared against one another to understand the intra-individual differences as measured from different keratin tissues through linear regression and a paired t-test analysis for pairwise comparisons.

To explore the possibility of predicting the geographical origin of individuals in a closed population, Classification, and Regression Tree (CART) decision tree models^51,52^ were developed. The classification tree is built by splitting the original data into smaller nodes (sub-partitions) based on a criterion that allows the nodes to become more homogeneous than the initial set. Then, through recursive partitioning, the dataset continues to split into smaller and smaller nodes and terminates once purity is reached for all sub-partitions. The CART decision tree model, a non-parametric supervised learning technique, was chosen over other methods as it allows for easy interpretation and enables non-experts to effectively utilize the tool^53^, which is an important consideration when developing tools for forensic applications.

Three CART models were trained using stable hydrogen and oxygen isotope data in hair as the predictor variables for Model 1, stable hydrogen and oxygen isotope values in toenails as the predictor variables for Model 2, and stable hydrogen and oxygen isotope values for both hair and toenails as the predictor variables for Model 3. Study sites 1–4 were set as the model response variables. The full dataset was partitioned into training and testing sets. Approximately 80% of the entire dataset was randomly selected as the training set to train the model (n = 65 for Model 1; n = 32 for Model 2; n = 28 for Model 3), and the remaining 20% were used as the test set to measure the performance of the classification model (n = 16 for Model 1; n = 7 for Model 2; n = 7 for Model 3). The decision tree model begins by splitting the training dataset into sub-datasets based on the best splitting attribute. The attributes are either the isotopic values of oxygen or hydrogen for Models 1 and 2, and oxygen or hydrogen values in hair or toenails for Model 3 that best classify the samples into one of the four study sites. The tree split points were determined by the Gini index ^54^, $Gini index=1-\sum_{i=1}^{j}{({p}_{i})}^{2}$, where *p* = proportion of misclassified observations. Gini index reaches zero as homogeneity increases for each node.

The model was evaluated for its accuracy by taking the sum of all true positives and true negatives over the sum of all values of a confusion matrix developed from the test set with a threshold value of 0.5 (Table 1):

**Table 1:** A 2 × 2 confusion matrix.

	Actual	
	Positive	Negative
**Predicted**		
Positive	True positive	False positive
Negative	False negative	True negative

$$Accuracy=\frac{\text{true positives}+{\text{true negatives}}}{{\text{true positivies}}+\text{ false positives}+\text{true negatives}+\text{false negatives}}$$

Other calculated measures include sensitivity, specificity, positive predictive value, and negative predictive value were also calculated to understand the performance of the models:

$$Sensitivity=\frac{{\text{true positives}}}{{\text{true positivies}}+\text{false negatives}}$$

$$Specificity=\frac{{\text{true negatives}}}{{\text{false positives}}+\text{true negatives}}$$

$$Positive predictive value=\frac{{\text{true positives}}}{{\text{true positivies}}+\text{false positives}}$$

$$Negative predictive value=\frac{{\text{true negatives}}}{{\text{true negatives}}+\text{false negatives}}$$

As can be seen from the equations, a high sensitivity value with a maximum of 1 indicates low false negatives, and a high specificity value indicates low false positives. In other words, sensitivity measures indicate the degree to which the predicted values were not from the predicted site. The positive predictive value, or precision, provides the ratio of the true positives for all predicted positives. The closer the measure is to 1, the higher the precision. The negative predictive value, on the other hand, provides the ratio of true negatives for all predicted negatives.

## Results

### The isotopic spread for each study site

**Figure 2:**
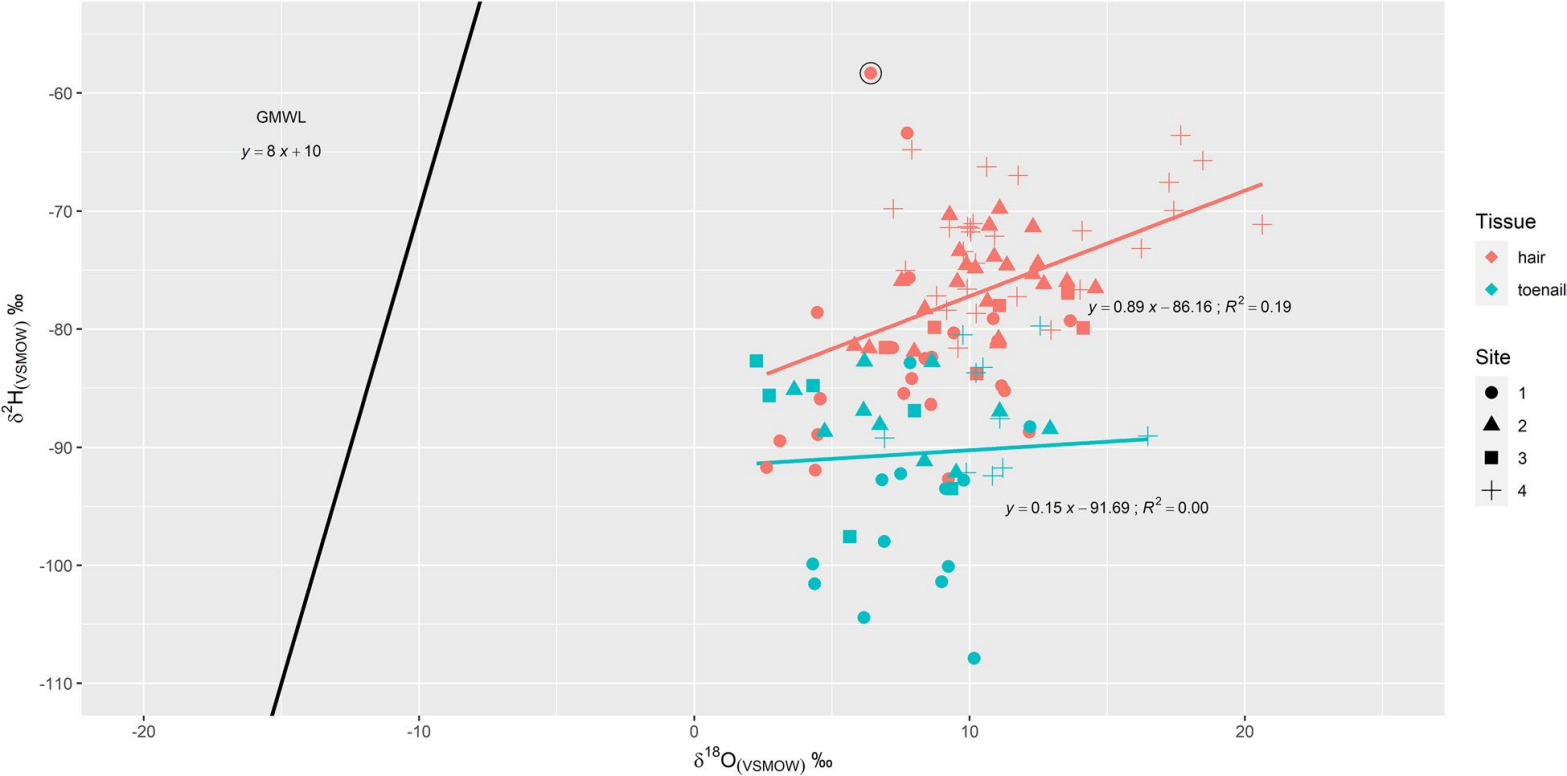
*δ*^2^H and *δ*^18^O values (‰) of all samples for both hair (*δ*^2^H: n = 81, *δ*^18^O: n = 82) and toenails (*δ*^2^H and *δ*^18^O: n = 39). The solid black line represents the Global Meteoric Water Line (GMWL) [*δ*^2^H = 8 $\times$ *δ*^18^O + 10] and is included in the graph for comparison purposes. The regression lines between oxygen and hydrogen values for hair [*δ*^2^H_hair(VSMOW)_ = 0.89 × *δ*^18^O_hair(VSMOW)_ − 86.16, R^2^ = 0.19, *p* < 0.01] and toenails [*δ*^2^H_toenail(VSMOW)_ = 0.15 × *δ*^18^O_toenail(VSMOW)_ − 91.69, R^2^ = 0.00, *p* = 0.69] are also indicated as solid lines in their respective colors. A statistical outlier as identified by measuring the Mahalanobis distance metric^55^ is marked by a black circle.

The overall linear relationship between *δ*^2^H and *δ*^18^O values for hair (n = 81) and toenails (n = 39), respectively, were (Fig. 2):

1 $$\delta^{2} {\text{H}}_{{\text{hair(VSMOW)}}} = \, 0.89 \times \delta^{18} {\text{O}}_{{\text{hair(VSMOW)}}} {-} \, 86.16,\;{\text{R}}^{2} = \, 0.19,\;p \, < \, 0.01$$

2 $$\delta^{2} {\text{H}}_{{\text{toenail(VSMOW)}}} = \, 0.15 \times \delta^{18} {\text{O}}_{{\text{toenail(VSMOW)}}} {-} \, 91.69,\;{\text{R}}^{2} = \, 0.00,\;p \, = \, 0.69$$

**Figure 3:**
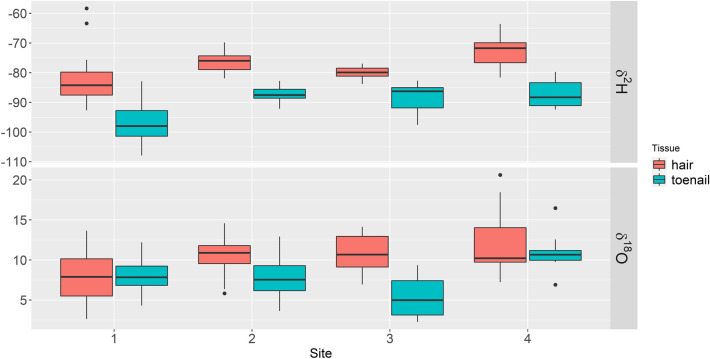
A box plot of hair and toenail data for each study site. Site 1: Vancouver, Site 2: Orillia, Site 3: Iqaluit, Site 4: Wolfville. The black dots represent outliers for each group of either *δ*^2^H or *δ*^18^O values however, differ from the statistical outliers when both *δ*^2^H or *δ*^18^O values are determined using the multivariate distance metric of Mahalanobis distance^55^.

*δ*^2^H and *δ*^18^O values in hair samples collected from individuals residing in Site 4 had the most positive values, and Site 1 had the most negative values (Table 2, Fig. 3). For toenails, the most positive *δ*^2^H and *δ*^18^O values were also observed for individuals from Site 4; however, the most negative *δ*^2^H and *δ*^18^O values were observed for Site 1 and Site 3, respectively. This is an interesting result as Site 3, Iqaluit, was expected to produce the lowest values for *δ*^2^H and *δ*^18^O values in both hair and toenails, given that it is located on a much higher latitude compared to the other sites, including Site 1 of Metro Vancouver (see Supplementary Fig. S1 online). However, such a pattern could not be observed for Iqaluit, except for *δ*^18^O values in toenails.

**Table 2:** Descriptive statistics of *δ*^2^H and *δ*^18^O in hair and toenails grouped by study sites.

*δ*^2^H							*δ*^18^O				
Site	n	Mean	Sd	Min	Max	Range	Mean	Sd	Min	Max	Range
**Hair**											
1	23	− 82.5	8.25	− 92.7	− 58.3	34.4	8.0	3	2.6	13.7	11.1
2	24	− 76.2	3.69	− 81.9	− 69.8	12.1	10.5	2.11	5.8	14.6	8.8
3	6	− 80.0	2.45	− 83.8	− 77.0	6.8	10.8	2.77	6.9	14.1	7.2
4	28	− 72.5	4.73	− 81.6	− 63.6	18.0	11.9	3.63	7.2	20.6	13.4
**Toenails**											
1	13	− 96.6	6.94	− 108.0	− 82.9	25.1	8.0	2.28	4.3	12.2	7.9
2	10	− 87.3	3.13	− 92.1	− 82.8	9.3	7.8	2.87	3.6	12.9	9.3
3	6	− 88.5	5.75	− 97.6	− 82.7	14.9	5.4	2.85	2.3	9.4	7.1
4	10	− 86.9	4.8	− 92.4	− 79.7	12.7	10.9	2.42	6.9	16.5	9.6

### MANOVA results (differences in δ^2^H and δ^18^O in tissues between study areas)

An outlier was detected for one hair sample (H2) with *δ*^2^H = − 58.3‰ and *δ*^18^O = 6.4‰ using the Mahalanobis distance ^55^. When MANOVA was run on the original dataset, including the outlier, mean *δ*^2^H and mean *δ*^18^O values differed between the four sampling sites as measured by the MANOVA test [F(3, 77) = 14.07, *p* < 0.01] and [F(3, 77) = 7.43, *p* < 0.01], respectively. MANOVA was also run on the dataset with the outlier removed but resulted in a similar result where *δ*^2^H and *δ*^18^O values of hair differed across the study sites at [F(3, 76) = 22.093, *p* < 0.01] and [F(3, 76) = 6.90, *p* < 0.01], respectively. Thus, the presence of the outlier did not influence the overall results for hair. Mean *δ*^2^H values and mean *δ*^18^O values in toenails differed between the four sampling sites as measured by the MANOVA test [F(3, 35) = 8.26, *p* < 0.01] and [F(3, 35) = 6.34, *p* < 0.01], respectively.

Results from the Tukey post-hoc test indicated that *δ*^2^H values in hair samples collected from Site 1 differed significantly from those collected from Sites 2 (*p* < 0.01) as well as from Site 4 (*p* < 0.01) (See Supplementary Table S2. online). Site 3, on the other hand, was more similar to Site 1; however, it was distinct from Site 4 (*p* < 0.05). Hair *δ*^18^O values also differed significantly between Sites 1 and 2, and between Sites 1 and 4. For toenails, samples from Site 1 showed *δ*^2^H values that were distinct from all other sites, whereas *δ*^18^O values in samples from Site 4 differed significantly from the other three sites.

### Human tissue and drinking water

*δ*^2^H and *δ*^18^O values of tap water from Wolfville, NS were − 61.8‰ and − 9.4‰, respectively. These data are also true for all cities in Site 4 as they are all supplied by the same Cornwallis watershed^42,43^. For the remaining study sites, isotopic data were collected from existing publications. Actual tap water data were retrieved for all cities in Site 1 from Ueda and Bell's^7^ extensive tap water study covering the entire regional district of Metro Vancouver, BC (Table 3). The tap water value for the city of Barrie, ON in Site 2, was downloaded from the Waterisotope Database^46^. Although the source water information was not provided, it was assumed that the data are sufficient as Barrie is mainly supplied by a single water source, the Kempenfelt Bay^29^, and thus has a high likelihood that this particular tap water sample came from the main drinking water source.

**Table 3:** *δ*^2^H and *δ*^18^O values of tap water for each location.

Site	Location	Tap water source	*δ*^2^H	*δ*^18^O	Type
1	Vancouver	Mixed capilano/seymour	− 89.0	− 11.7	Tap^4^
1	Burnaby	Capilano/seymour	− 89.0	− 11.1	Tap^4^
1	Surrey	Seymour/coquitlam	− 87.3	− 11.3	Tap^4^
1	Port moody	Coquitlam	− 86.2	− 11.1	Tap^4^
1	Coquitlam	Coquitlam	− 86.2	− 11.1	Tap^4^
1	New westminster	Seymour/coquitlam	− 86.8	− 11.2	Tap^4^
1	Richmond	Capilano/seymour	− 88.1	− 11.6	Tap^4^
2	Orillia	Lake couchiching + two ground water sources (wells 1 and 2)^28^	− 64.2	− 9.0	Surface water (Black River near Washago)^56^
2	Barrie	Kempenfelt bay^29^	− 82.2	− 12.0	Tap^46^
2	Horseshoe valley	Ground water^32^	− 77.0	− 11.1	OIPC^47^
2	Washago	Lake couchiching^30^	− 64.2	− 9.0	Surface water (Black river near Washago)^56^
2	Lafontaine	Ground water (severn sound watershed)^33^	− 77.0	− 11.1	OIPC^47^
2	Gravenhurst	Lake muskoka^31^	− 69.7	− 9.5	Surface water^57^
2	Midland	Ground water (severn sound watershed)^34^	− 76.0	− 11.1	OIPC^47^
3	Iqaluit	Lake geraldine^36^	− 129.0	− 17.3	OIPC^47^
4	Wolfville	Ground water (cornwallis watershed)^42,43^	− 61.8	− 9.4	Tap (Current study)
4	Kentville	Ground water (cornwallis watershed)^42,43^	− 61.8	− 9.4	Tap (Current study)
4	Berwick	Ground water wells (cornwallis watershed)^41,43^	− 61.8	− 9.4	Tap (Current study)

Surface water data were retrieved for locations where actual tap water data could not be found. Isotopic data of the Black River near Washago, ON^56^ were chosen as the most representative data for Lake Couchiching as no isotopic data on water samples collected directly from Lake Couchiching were found. Another city in Site 2, Gravenhurst, ON, primarily draws drinking water from Lake Muskoka. Untreated water from Lake Muskoka is drawn proximate to Brydon's Bay for treatment and distribution to residents across Gravenhurst. Thus data from the drainage area identified as MA3BA in James et al.'s^57^ study, which covered the entire area of Brydon's Bay, was determined to best represent drinking water isotope values for Gravenhurst. Finally, the Online Isotope Precipitation Calculator was used to estimate drinking water values for the remaining cities of Horseshoe Valley, ON and Midland, ON in Site 2, and Iqaluit, NU in Site 3.

**Figure 4:**
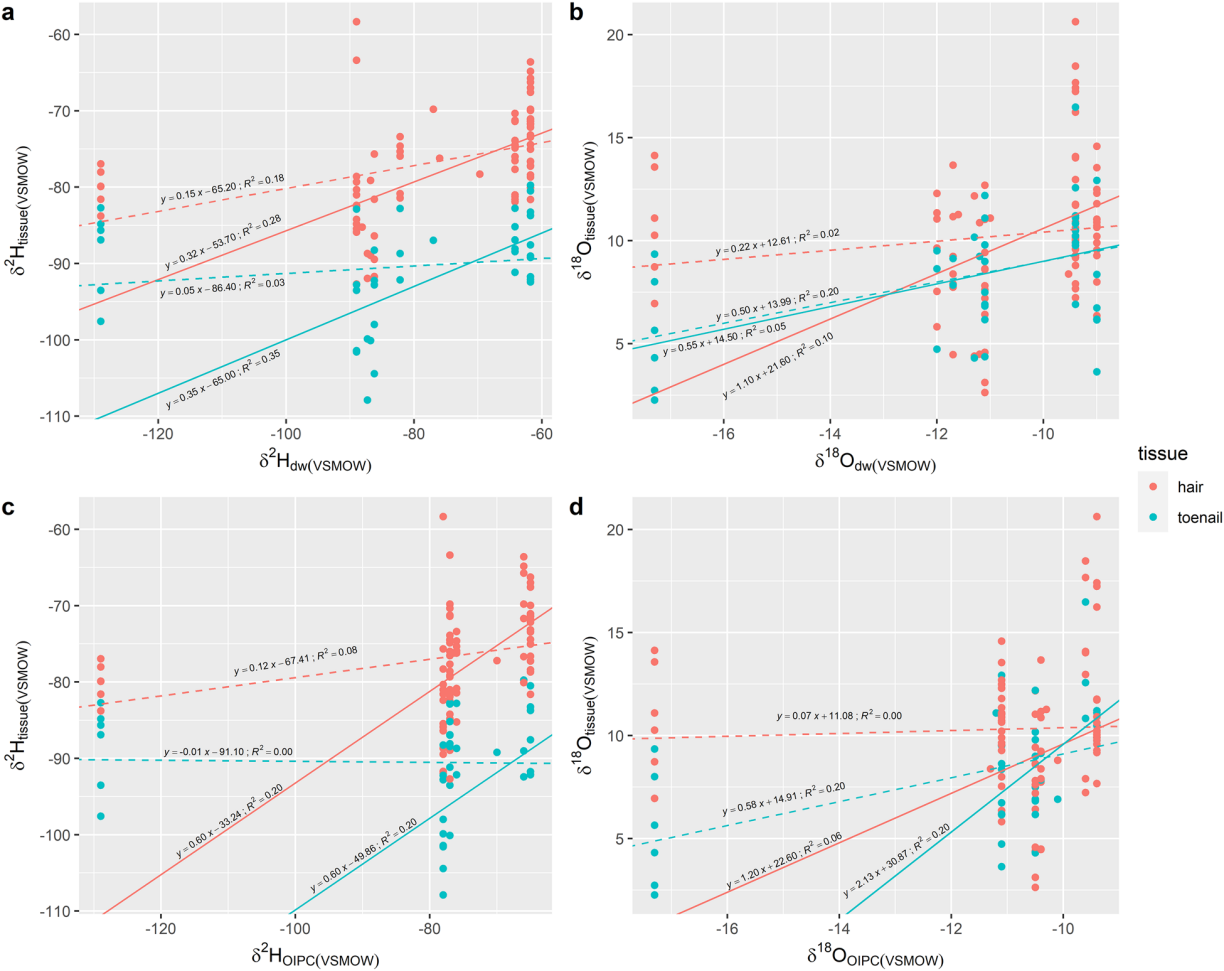
A plot of (**a**) *δ*^2^H values and (**b**) *δ*^18^O values for human tissues and local drinking water and (**c**) *δ*^2^H values and (**d**) *δ*^18^O values for human tissues and precipitation data as obtained from the Online isotope precipitation calculator^58^. Colours indicate tissue type. Lines represent the linear relationships between the stable isotope compositions of human tissues and drinking water. The dotted lines show the relationships for all samples, and the solid lines show relationships for all samples excluding those from Iqaluit. These samples were treated as outliers, given that stable hydrogen and oxygen isotope values of tissue samples from Iqaluit were significantly higher than expected, and local drinking water values were those estimated using the Online Isotope Precipitation Calculator^58^ rather than those of Iqaluit tap water samples. The exclusion of Iqaluit samples generally increased the R^2^ value for most equations.

The retrieval of drinking water isotope data allowed the determination of the relationship between stable hydrogen and oxygen isotopes in modern human tissues and drinking water. The linear regression results between *δ*^2^H of hair (*δ*^2^H_hair_) and drinking water (*δ*^2^H_dw_) for all study sites with drinking water values (n = 81) were (Fig. 4):

3 $$\delta^{2} {\text{H}}_{{{\text{hair}}}} = \, 0.15 \times \delta^{2} {\text{H}}_{{{\text{dw}}}} {-} \, 65.17,\;{\text{R}}^{2} = \, 0.18,\;p \, < \, 0.01$$

*δ*^2^H of toenails (*δ*^2^H_toenails_) and drinking water gave a relationship of:

4 $$\delta^{2} {\text{H}}_{{{\text{toenails}}}} = \, 0.05 \times \delta^{2} {\text{H}}_{{{\text{dw}}}} {-} \, 86.40,\;{\text{R}}^{2} = \, 0.03,\;p \, = \, 0.32$$

*δ*^18^O of hair (*δ*^18^O_hair_) and drinking water (*δ*^18^O_dw_):

5 $$\delta^{18} {\text{O}}_{{{\text{hair}}}} = \, 0.20 \times \delta^{18} {\text{O}}_{{{\text{dw}}}} + \, 12.61,\;{\text{R}}^{2} = \, 0.02,\;p \, = \, 0.21$$

And finally, *δ*^18^O of toenails (*δ*^18^O_toenails_) and drinking water

6 $$\delta^{18} {\text{O}}_{{{\text{toenails}}}} = \, 0.50 \times \delta^{18} {\text{O}}_{{{\text{dw}}}} + \, 13.99,\;{\text{R}}^{2} = \, 0.20,\;p \, < \, 0.05$$

Tissue values from all study sites were also regressed against OIPC values that were calculated from latitudinal, longitudinal, and altitude data (Fig. 4):

7 $$\delta^{2} {\text{H}}_{{{\text{hair}}}} = \, 0.12 \times \delta^{2} {\text{H}}_{{{\text{OIPC}}}} {-} \, 67.41,\;{\text{R}}^{2} = \, 0.08,\;p \, < \, 0.01$$

8 $$\delta^{2} {\text{H}}_{{{\text{toenails}}}} = \, {-}0.01 \times \delta^{2} {\text{H}}_{{{\text{OIPC}}}} {-} \, 91.10,\;{\text{R}}^{2} = \, 0.00,\;p \, = \, 0.89$$

9 $$\delta^{18} {\text{O}}_{{{\text{hair}}}} = \, 0.07 \times \delta^{18} {\text{O}}_{{{\text{OIPC}}}} + \, 11.08,\;{\text{R}}^{2} = \, 0.00,\;p \, = \, 0.70$$

10 $$\delta^{18} {\text{O}}_{{{\text{toenails}}}} = \, 0.58 \times \delta^{18} {\text{O}}_{{{\text{OIPC}}}} + \, 14.91,\;{\text{R}}^{2} = \, 0.24,\;p \, < \, 0.01$$

Actual tap water data could not be retrieved for Iqaluit and given that higher-than-expected stable hydrogen and oxygen isotope values were measured for individuals from Iqaluit, these samples were removed as it was unclear whether the OIPC generated values were appropriate estimates for Iqaluit drinking water. Below are the relationships between human tissue values and drinking water values where Iqaluit samples were excluded from the analysis (Fig. 4).

11 $$\delta^{2} {\text{H}}_{{{\text{hair}}}} = \, 0.32 \times \delta^{2} {\text{H}}_{{{\text{dw}}}} {-} \, 53.69,\;{\text{R}}^{2} = \, 0.28,\;p \, < \, 0.01$$

12 $$\delta^{2} {\text{H}}_{{{\text{toenails}}}} = \, 0.35 \times \delta^{2} {\text{H}}_{{{\text{dw}}}} {-} \, 64.99,\;{\text{R}}^{2} = \, 0.35,\;p \, < \, 0.01$$

13 $$\delta^{18} {\text{O}}_{{{\text{hair}}}} = \, 1.10 \times \delta^{18} {\text{O}}_{{{\text{dw}}}} + \, 21.57,\;{\text{R}}^{2} = \, 0.1,\;p \, < \, 0.01$$

14 $$\delta^{18} {\text{O}}_{{{\text{toenails}}}} = \, 0.55 \times \delta^{18} {\text{O}}_{{{\text{dw}}}} + \, 14.53,\;{\text{R}}^{2} = \, 0.05,\;p \, = \, 0.22$$

And for OIPC values (Fig. 4):

15 $$\delta^{2} {\text{H}}_{{{\text{hair}}}} = \, 0.60 \times \delta^{2} {\text{H}}_{{{\text{OIPC}}}} {-} \, 33.24,\;{\text{R}}^{2} = \, 0.2,\;p \, < \, 0.01$$

16 $$\delta^{2} {\text{H}}_{{{\text{toenails}}}} = \, 0.60 \times \delta^{2} {\text{H}}_{{{\text{OIPC}}}} {-} \, 49.86,\;{\text{R}}^{2} = \, 0.2,\;p \, < \, 0.05$$

17 $$\delta^{18} {\text{O}}_{{{\text{hair}}}} = \, 1.20 \times \delta^{18} {\text{O}}_{{{\text{OIPC}}}} + \, 22.59,\;{\text{R}}^{2} = \, 0.06,\;p \, < \, 0.05$$

18 $$\delta^{18} {\text{O}}_{{{\text{toenails}}}} = \, 2.13 \times \delta^{18} {\text{O}}_{{{\text{OIPC}}}} + \, 30.87,\;{\text{R}}^{2} = \, 0.2,\;p \, < \, 0.01$$

The R^2^ values increased for all four equations when Iqaluit samples were removed. Overall, stable hydrogen and oxygen isotope values in modern human tissues are weakly related to those in drinking water as measured by tap water. The R^2^ value showed a slight increase for *δ*^18^O values in toenails when the OIPC precipitation data were used instead of tap and surface water values; however still on the lower end at R^2^ = 0.2. Therefore, there is no clear evidence from this dataset that a latitudinal gradient exists for hydrogen and oxygen values in human tissues for Canada as measured by the stable isotope values of drinking water.

### The CART model

**Figure 5:**
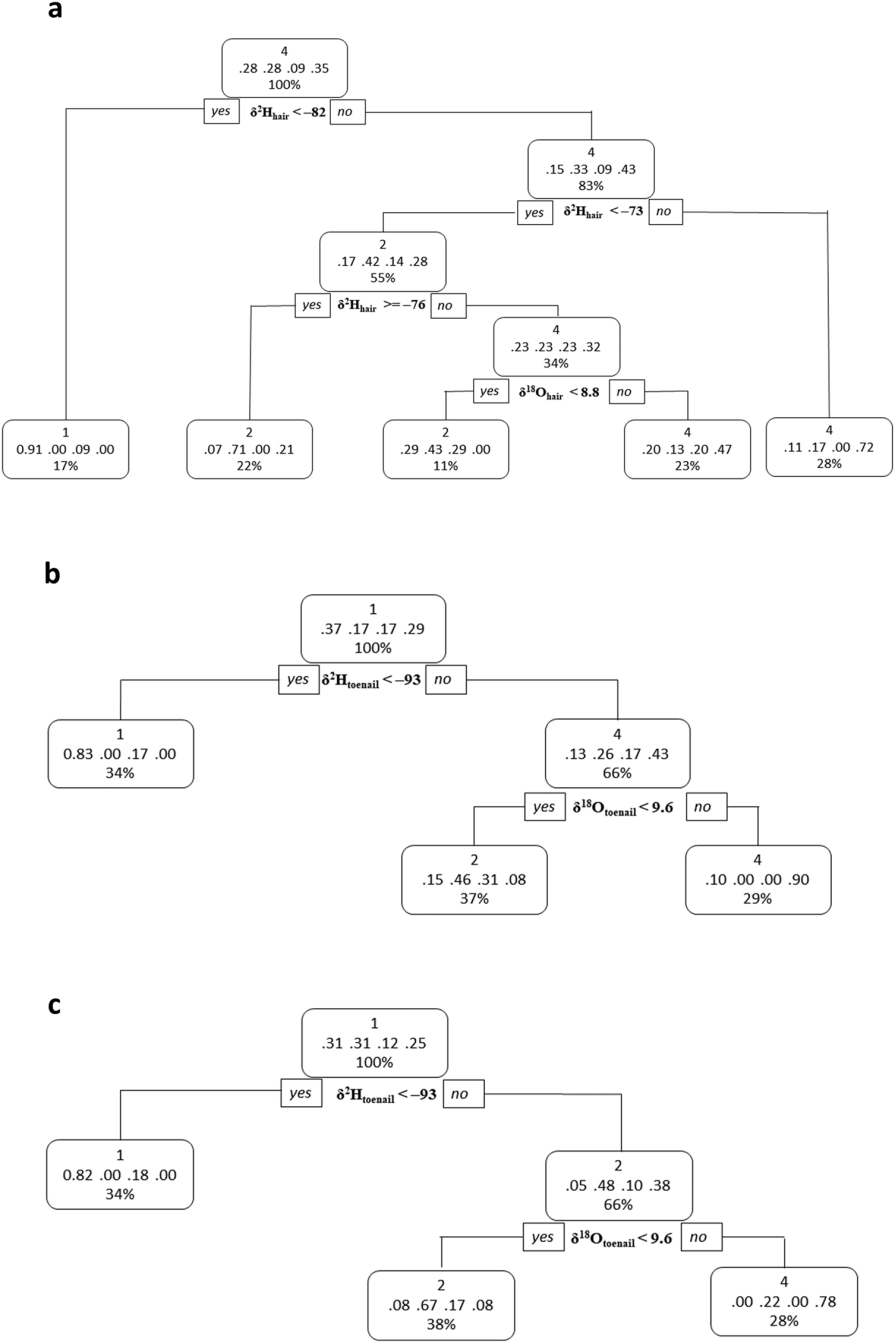
Decision trees developed from both *δ*^2^H and *δ*^18^O values of (**a**) hair [Model 1, trained with n = 65], (**b**) toenails [Model 2, trained with n = 32] and (**c**) of both hair and toenails [Model 3, trained with n = 28]. The predicted study site numbers are shown on the first row within each bubble. The proportions of samples in each node are shown as decimals for Sites 1, 2, 3, 4, respectively. The percentages indicate the proportion of samples within each sub-partition.

The first CART decision tree model was built for hair samples with stable hydrogen and oxygen isotope values (Model 1) (Fig. 5a). The split cut-off for node 1 was determined by hydrogen values where any samples with *δ*^2^H_hair_ values less than − 82‰ were predicted as Site 1. Samples with *δ*^2^H_hair_ > − 82‰ were then split further where any samples with *δ*^2^H_hair_ values less than − 73‰ were initially classified as Site 2. These samples were then split again to either Site 2 (*δ*^2^H_hair_ ≥ 76‰) or Site 4 (*δ*^2^H_hair_ < 76‰). Hair samples classified as Site 4 at this stage were further classified into Site 2 or 4 depending next on the *δ*^18^O values. Finally, all samples with *δ*^2^H_hair_ > − 73‰ were classified as Site 4. No samples could be classified as originating from Site 3. The second CART model was built for stable hydrogen and oxygen isotopes of toenails (Model 2) (Fig. 5b). The model included only two decision nodes in which the first predictor variable was *δ*^2^H_toenail_ value, where samples with values less than − 93‰ were predicted to be from Site 1. For toenail samples with hydrogen values greater than − 93‰, oxygen values were used to determine whether they could be classified as Site 2 or Site 4. Those samples with *δ*^18^O_toenail_ values less than 9.6‰ were classified as Site 2 and those with values greater than 9.6‰ were predicted as Site 4. No samples were predicted to be from Site 3 purely from stable hydrogen and oxygen isotopes in toenails. Finally, the third model consisted of stable hydrogen and oxygen isotope values in both hair and toenail samples (Model 3) (Fig. 5c). Model 3 selected toenails as the best attribute for classification, which indicates that toenail isotope values are the better predictor when both hair and toenail samples are present for analysis from Sites 1–4. The model was similar to that of Model 2.

Confusion matrices (Table 1) were constructed for all three models to evaluate the performance of the classification models. Of the three models, Model 3 proved to be the most accurate model with an overall accuracy of 71.4% (see Supplementary Fig S2. online). The performance evaluation summary, including measures for sensitivity, specificity, positive predictive value, and negative predictive value for all three models, is provided in (see Supplementary Table S3. online).

### Intra-individual differences

**Figure 6:**
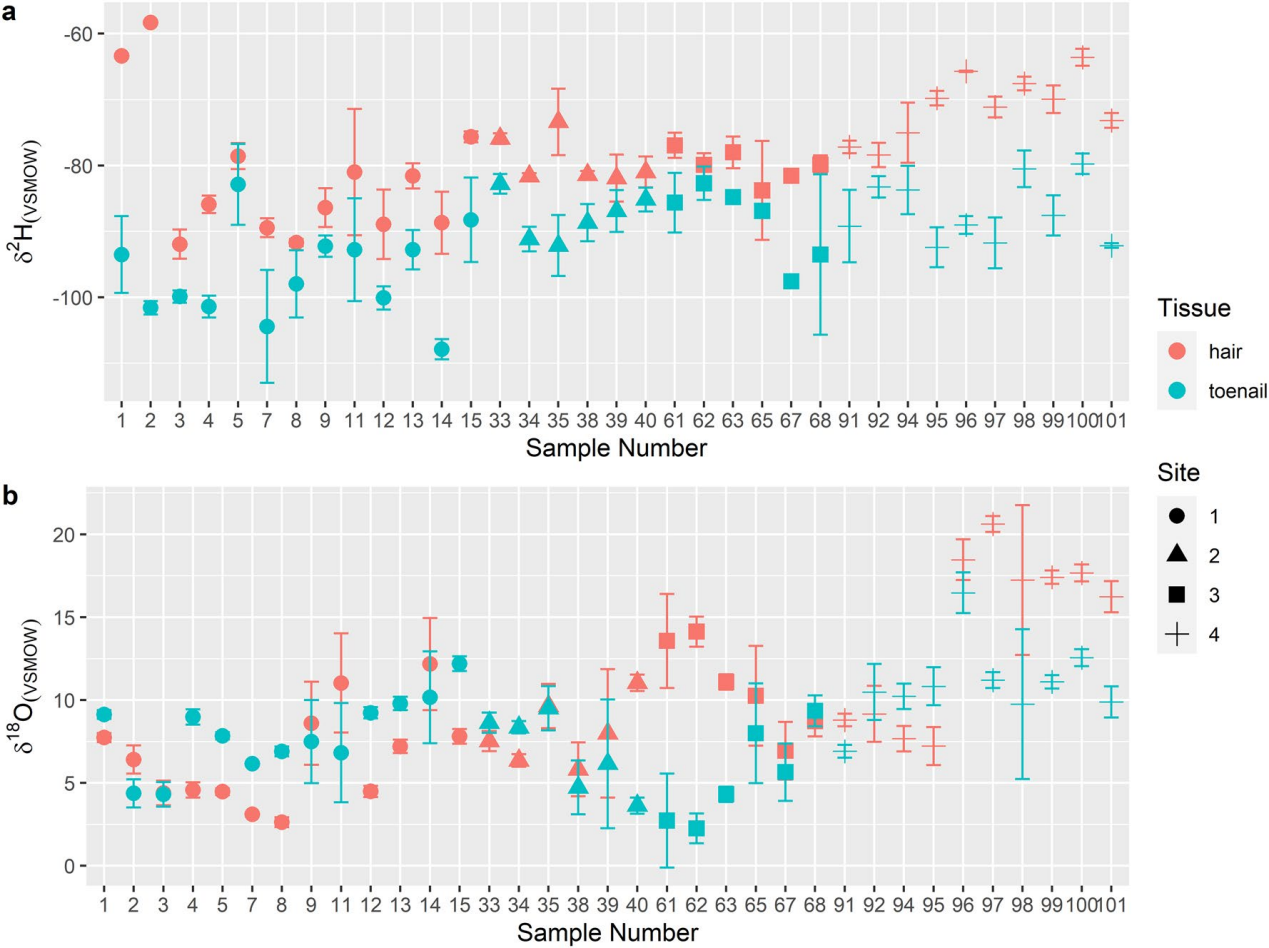
(**a**) *δ*^2^H and (**b**) *δ*^18^O values in hair and toenails for all individuals that provided both tissue types (n = 35). Study site information are also shown by shapes. The standard deviations of each sample, ran in either duplicates or triplicates, are shown by error bars. Note that error bars cannot be seen for some samples due to small standard deviations. The average difference between the isotopic values of hair and toenail from the same individual were 13.0‰ with a standard deviation of 8.4‰ for *δ*^2^H and 1.5‰ with a standard deviation of 4.6‰ for *δ*^18^O.

Both hair and toenail samples were retrieved from 35 of the 86 individuals. The paired difference between *δ*^2^H values in hair and toenails of the same individual was tested using the Wilcoxon Signed Rank's test for non-normal data as the dataset failed the Shapiro–Wilk's normality test at the α = 0.05 significance level. Significant differences were found between *δ*^2^H values of hair (n = 35, mean = − 78.0‰, s.d. = 3.06) and toenails (n = 35, mean = − 90.9‰, s.d. = 3.27) from the same individual (*p* < 0.05). The paired t-test was utilized to assess for similarities in *δ*^18^O values of hair and toenail from the same individual as the dataset was normally distributed. The results indicated that there were no significant differences between *δ*^18^O values of hair (n = 35, mean = 9.7‰, s.d. = 1.22) and toenails (n = 35, mean = 8.2‰, s.d. = 2.10) at the 0.05 significance level, t(34) = 1.92, *p* > 0.05. Overall, the isotopic values of *δ*^2^H in hair were higher than those of toenail from the same individual by 13.0‰, on average, with a standard deviation of 8.4‰. For *δ*^18^O, the average was 1.5‰ with a standard deviation of 4.6‰ (Fig. 6).

The linear relationships between *δ*^2^H in hair and toenails for all individuals were (see Supplementary Fig S3. online):

19 $$\delta^{2} {\text{H}}_{{{\text{hair}}}} = \, 0.48 \times \delta^{2} {\text{H}}_{{\text{toenail }}} {-} \, 34.72,\;{\text{R}}^{2} = \, 0.16,\;p \, < \, 0.05$$

and for *δ*^18^O:

20 $$\delta^{18} {\text{O}}_{{{\text{hair}}}} = \, 0.55 \times \delta^{18} {\text{O}}_{{{\text{toenail}}}} + \, 5.16,\;{\text{R}}^{2} = \, 0.13,\;p \, < 0.05$$

Overall, both equations showed a weak relationship, as seen by the small R^2^ values.

## Discussion

### Hydrogen and oxygen in human tissues

Stable isotope compositions of hydrogen and oxygen in human hair and toenails overall showed overlap across the different study sites selected for this study. Given that Iqaluit is located higher in latitude compared to other study sites, the hydrogen and oxygen values of tissues from individuals residing in Iqaluit were expected to show the most negative values. The estimated local precipitation value for Iqaluit was the lowest amongst the four study sites (Table 3); however, stable isotope values of both hair and toenail samples from Iqaluit showed significant overlap with samples from Sites 1, 2, and 4. A similar trend has also been observed by Bowen et al.^6^ for hair in Inuit populations, where hydrogen values of Inuit hair were reported to be higher than expected and even consistent with those from tropical climates. This was possibly due to some influence of a marine-based diet on hydrogen isotope values of hair as the hydrogen values can be influenced by the amount and type of meat consumed^1,6^ and this may also be true for Iqaluit individuals. Another plausible explanation may be that individuals from Iqaluit were consuming isotopically enriched water. The community is regularly under water advisories due to inadequate infrastructure in the community leading to the contamination of drinking water sources^37^ and thus the community may rely on transported bottled water with potentially higher isotope values^59^.

Samples from Site 4 generally gave the most positive stable hydrogen and oxygen isotope values in hair, and oxygen values in toenails. Such findings were as expected given the relatively higher isotope values for tap water supplying the area. Wolfville and its surrounding cities rely on ground water sources (Table 3) and the higher values may be influenced by the maritime weather system on local precipitation values^43^. The exception was with hydrogen values of toenails from Site 4, which overlapped significantly with those from Site 2, Orillia. Site 2, is located adjacent to the Great Lakes, and multiple water sources supply the area. Some regions rely on surface water systems, whereas others rely on groundwater systems. Hydrogen and oxygen values for the ground water sources are similar to those of drinking water values for Site 4 and thus may explain the overlap in values observed for Sites 2 and 4. Finally, the general trend observed for Sites 1, 2 and 4 followed the general pattern shown in Chartrand and St-Jean^59^’s report on Canadian hair.

Stable hydrogen and oxygen isotope compositions of human keratinous tissues such as hair and nails have been shown to reflect local precipitation water values, with previous work suggesting that drinking water isotopes are more closely reflected in hair than in nails^14,16^. Results from this study (Fig. 2) similarly indicated that stable hydrogen and oxygen isotopes are more closely reflected in hair than in toenails. The slopes for both hair and toenails were lower than that of the Global Meteoric Water Line (GMWL), a linear relationship between hydrogen and oxygen of meteoric water, and were consistent with existing studies^14,16,22,59^. The low correlation between hydrogen and oxygen values in hair, as measured by the r-squared metric, was similarly observed by Mant et al.’s^22^ study perhaps due to the differences in the sources of hydrogen and oxygen that are reflected in human tissues, where hydrogen values are more influenced by dietary input compared to oxygen^3,18^.

### Human tissue and drinking water

#### Hair

The slope of the regression line between the stable isotope values in hair and drinking water has been interpreted as the proportion in which isotope values in drinking water are preserved in human hair. The slope is generally expected to be less than one^3,14,22,59^ and similar observations were seen for this study. Correlation, as measured by the R^2^ value, was low however, and indicates that the linear model does not fully explain the relationship between the two variables. The low correlation may be a result of intrapopulation variation that is not fully explained by drinking water values alone, even with the incorporation of actual tap water data. Such an issue may be more present in larger conurbations where residents rely on multiple tap water sources with complex water distribution systems. The decrease in the correlation for local precipitation values compared to tap water values may further indicate that actual drinking water values should be used over estimated precipitation values for validating the relationship between drinking water and human tissues. This is further supported by the increase in the r-squared values of the linear regression models with the removal of Iqaluit samples that were paired with OIPC values (Fig. 4). Overall, the linear models produced low r-squared values and indicate that more validation work should be conducted before applying such models to forensic cases. Additionally, *δ*^2^H values in human hair have recently been demonstrated to be influenced by post mortem exposure to environmental conditions with values showing increases with time ^60^. Such a finding further highlights the need to re-investigate the use of water-to-tissue regression models especially for forensic geolocation.

#### Toenail

The slopes for the linear relationships between toenails and water values for hydrogen were the same as that observed for hair (Eq. 12) where Iqaluit samples were excluded from the calculation. The comparable slope observed for both hair and toenails suggests that drinking water hydrogen is incorporated into the tissues through similar processes. The slope of the equation (Eq. 12) was also comparable to the nail and drinking water equation reported by Fraser and Meier-Augenstein^14^, where their slope was reported to be 0.37. A similar slope was also reported by Mancuso and Ehleringer^13^, with a slope of 0.32. This slope increased further to 0.6 (Eq. 8) when the toenail values were compared against OIPC-generated local precipitation values, although a lower correlation of 0.2 indicates a weaker correlation with toenail values compared to hair values. Given the result, it is difficult to ascertain whether a linear model can appropriately explain the relationship between stable oxygen isotope compositions of toenails and drinking water in this sample.

### Intra-individual differences

The higher isotopic values observed for stable hydrogen isotopes in hair compared to toenails from the same individual for this study (13‰) fell within the range reported by Lehn et al.’s^15^ paper of 7–14‰ based on three individuals. The authors also noted that the hydrogen values of hair decrease with time where hair formed longer than 6 months were only 2–5‰ higher than nails. The standard deviation of hair to toenail differences observed for this study was 8.4‰, thus could be a result of hair that was sampled at varying lengths. Because hair and toenails have different growth rates, it can be difficult to ensure that the distal ends of the toenails were formed during the same time period or environment as the hair samples. Hence the direct comparison of isotope values for the tissues should be made with some caution particularly for stable hydrogen isotopes. However, for the oxygen values in hair and toenails from the same individual did not show any obvious differences. This may indicate that the two tissues were in fact formed in similar environmental conditions. Thus, the offset observed for hydrogen may be a result of differences in physiological processes and the incorporation of hydrogen into the respective tissues^14,15^.

### Within-site variation

Stable isotope compositions of hydrogen and oxygen values between individuals naturally show variation even between those from the same geographical locale. The understanding of such variation is important for stable isotope studies, especially when developing valid and accurate models based on known samples as it could inform the collection process, such as the number of samples needed to capture the entire range of isotope values for that region. The largest intrapopulation variations for hydrogen values were observed for Site 1 for both hair and toenails, and Site 4 for oxygen in both hair and toenails. All ranges are of those without removing the Iqaluit samples. The large variation cannot be fully explained by drinking water values alone where the linear regression between drinking water and tissue values produced low r-squared values and indicate that further research is necessary to understand other possible explanations for the large variations. This is particularly evident for Site 1 where there is an extensive knowledge of tap water stable isotope values for the region making it possible to confidently compare human tissue and drinking water values. Hydrogen values in tap water for Site 1 had range of 2.8 ‰ for hydrogen (Table 3), which was far lower than the range for hair hydrogen values from the same site. Although there is an overall difference between the study sites, the large intrapopulation variation indicates that a single sample cannot fully represent an entire geographical region.

### The CART model

The CART model, which includes models such as decision trees, can classify data into a specified number of groups using certain variables. This study investigated four distinct geographical areas across Canada and explored the possibility of using a classification model to group individuals according to their stable hydrogen and oxygen isotopes in human hair and toenails. Of the three models developed, Model 3 with both hair and toenail data as variables, had the highest accuracy, indicating that the model worked particularly well for our dataset. The best classifier for this model was toenail hydrogen, which can be explained by the difference in values observed for Site 1 compared to the other study sites (see Supplementary Table S2. online). The second classifier for Model 3 was toenail oxygen and can also be explained by the differences observed for toenail oxygen values between Sites 4 and 2 and between Sites 4 and 3. While the CART decision tree model is simplistic, the advantages are apparent in that it is intuitive and can be utilized by those who may not necessarily have the expertise to handle complex models. The tree can also be used to identify the most important variable that can help with the classification of individuals. For a closed population of individuals from four distinct study sites across Canada—Vancouver, Iqaluit, Wolfville, and Oakville—stable hydrogen and oxygen isotopes in toenails can be used to classify these individuals with an accuracy of up to 71.4%. All models were particularly good at classifying Vancouver samples (Site 1) compared to other study sites and thus indicate that stable hydrogen and oxygen isotopes of human hair and toenails could provide useful geographical information for a closed population. Overall, the performance of a CART approach offers a promising new way to refine stable isotope geolocation studies.

## Conclusion

Stable isotope values of hair and toenail samples, when compared against local drinking water values, showed low correlations indicating that the linear model does not fully explain the variation observed for hydrogen and oxygen values in this study. Furthermore, the stable isotope values of tissue samples from Iqaluit were higher than expected and confirmed the importance of obtaining local drinking water samples over estimating local precipitation values, as suggested by Ueda and Bell^7^. Results for Vancouver also showed intr-apopulation variation as high as 34.4‰ for hydrogen and 25.1‰ for oxygen. Such large variations could not be explained by drinking water values alone. If regression models are to be developed further for predictive analysis, multiple known tissue samples should be collected from each study site to ensure that intra-population variation is captured for that study site. Furthermore, the exploration into the use of CART models proved to be a promising approach for identifying a closed group of unknown individuals who are known to have resided in a limited number of geographical regions. The CART model will allow investigators to interpret human tissue isotope delta values to obtain geographical information of unknown individuals, without the need to apply complex mathematical models, and should be explored further as a new tool to assist with the identification of unknown human remains.

## Supplementary Information

Supplementary Information.

## Data Availability

All data analysed during the current study are available from the corresponding authors on reasonable request.
