# Supplementary material for: The application of a CART model for forensic human geolocation using stable hydrogen and oxygen isotopes
Source: Sci Rep. 2022 Dec 7;12:21169. doi: 10.1038/s41598-022-25394-w (PMC9729567; doi:10.1038/s41598-022-25394-w)
Supplement: Supplementary file 1 — Supplementary Information. [file 41598_2022_25394_MOESM1_ESM.pdf]

Title: The application of a CART model for forensic human geolocation using stable hydrogen and oxygen isotopes

Momoko Ueda[1], Lynne S. Bell[1*]

[1] School of Criminology, Centre for Forensic Research, Simon Fraser University, Burnaby, British Columbia, Canada

* Corresponding author

E-mail: lynneb@sfu.ca (LSB)

FIGURES

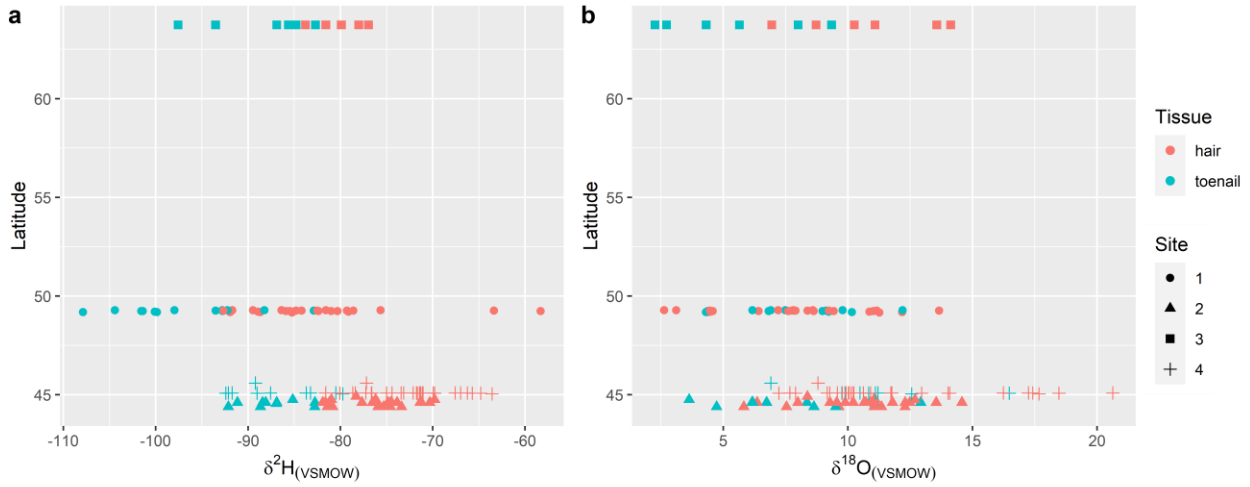

Figure S1. A scatterplot of a) δ^2H and b) δ^{18}O values of both hair and toenails by latitude. Study sites are shown by the shapes and tissue types by color. No apparent latitudinal influence was observed for both stable hydrogen and oxygens isotope compositions of human hair and toenails.

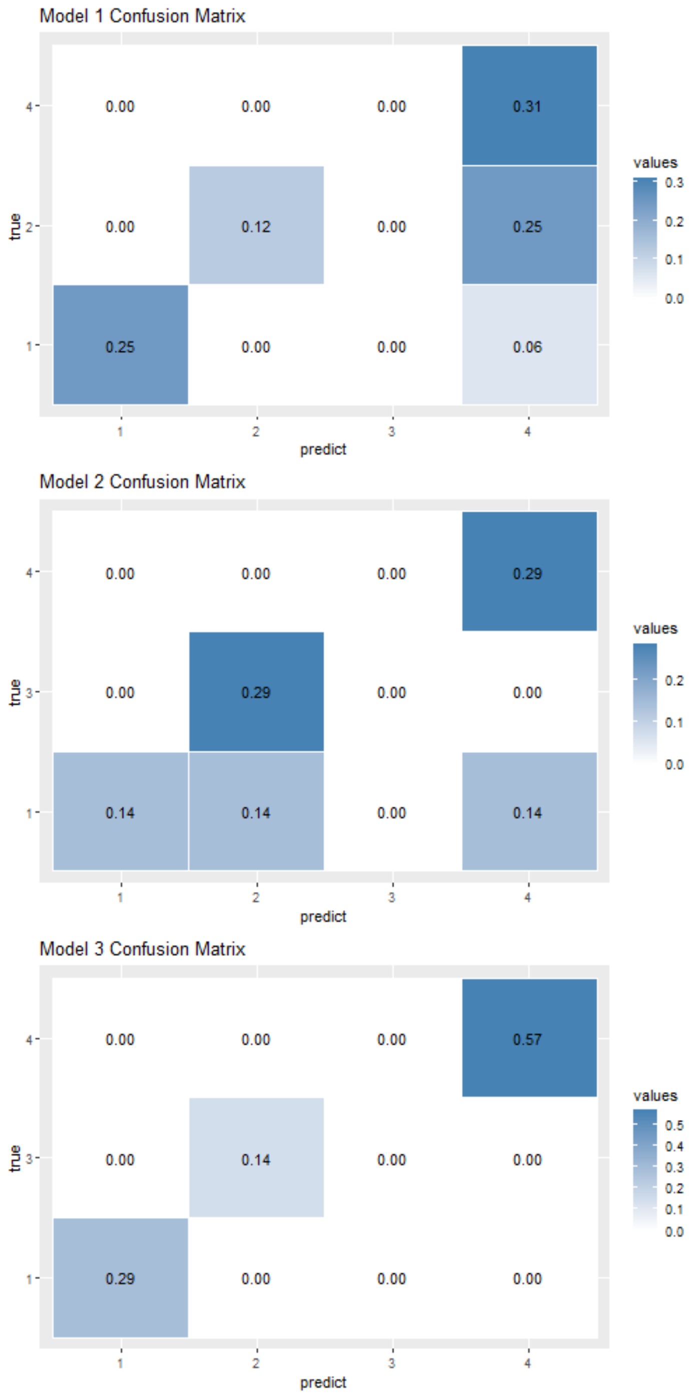

Figure S2. Confusion matrices of Models 1, 2 and 3 comparing model-predicted values with actual values. Numbers indicate the percentage of samples falling within each cell. Of the three models, Model 3 proved to be the most accurate model with an overall accuracy of 71.4%. Models 1 and 2 had accuracies of 50% and 42.9%, respectively.

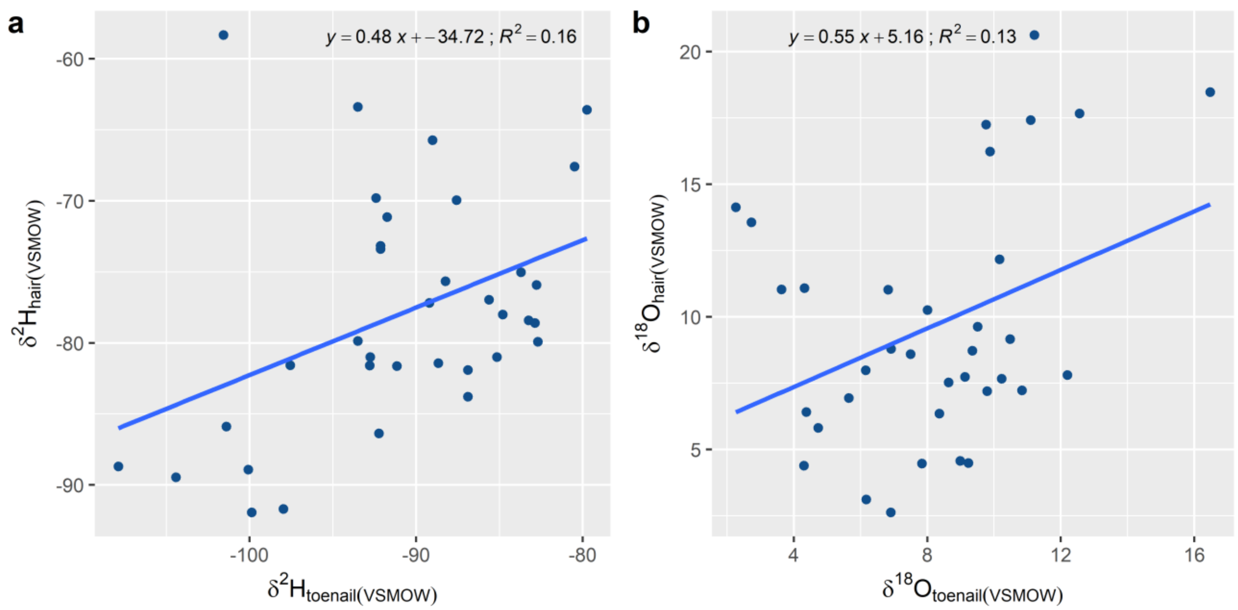

Figure S3. A plot showing the linear relationship between hair and toenails for a) δ^2H [δ^2H_{hair} = 0.48 × $\delta^2H_{toenail}$ – 34.72, R^2 = 0.16, $p < 0.05$] and b) δ^{18}O [$\delta^{18}O_{hair}$ = 0.55 × $\delta^{18}O_{toenail}$ + 5.16, R^2 = 0.13, $p < 0.05$] values (n=35). The low R^2 values of both equations signify the weak relationships between stable isotope values of hair and toenails from the same individual.

TABLES

Table S1. δ^2H and δ^{18}O data of all collected samples.

Sample ID	Site	Location	δ^2H	δ^{18}O	Tissue
H1	1	Vancouver	–63.4	7.7	hair
T1	1	Vancouver	–93.5	9.1	toenail
T2	1	Burnaby	–101.6	4.4	toenail
H2*	1	Burnaby	–58.3	6.4	hair
H3	1	Surrey	–91.9	4.4	hair
T3	1	Surrey	–99.9	4.3	toenail
H4	1	Burnaby	–85.9	4.6	hair
T4	1	Burnaby	–101.4	9.0	toenail
H5	1	Vancouver	–78.6	4.5	hair
T5	1	Vancouver	–82.9	7.8	toenail
T7	1	Port Moody	–104.4	6.2	toenail
H7	1	Port Moody	–89.5	3.1	hair
H8	1	Coquitlam	–91.7	2.6	hair
T8	1	Coquitlam	–98.0	6.9	toenail
H9	1	Coquitlam	–86.4	8.6	hair
T9	1	Coquitlam	–92.2	7.5	toenail
H11	1	Burnaby	–81.0	11.0	hair
T11	1	Burnaby	–92.8	6.8	toenail
H12	1	New Westminster	–88.9	4.5	hair
T12	1	New Westminster	–100.1	9.2	toenail
H13	1	Coquitlam	–81.6	7.2	hair
T13	1	Coquitlam	–92.8	9.8	toenail
H14	1	Surrey	–88.7	12.2	hair
T14	1	Surrey	–107.9	10.2	toenail
T15	1	Coquitlam	–88.2	12.2	toenail
H15	1	Coquitlam	–75.7	7.8	hair
H16	1	Vancouver	–92.7	9.2	hair
H18	1	Burnaby	–80.3	9.4	hair

Sample ID	Site	Location	δ^2H	δ^{18}O	Tissue
H19	1	Vancouver	–79.3	13.7	hair
H21	1	Vancouver	–84.8	11.2	hair
H22	1	Richmond	–85.2	11.3	hair
H23	1	New Westminster	–79.1	10.9	hair
H24	1	Vancouver	–82.5	8.4	hair
H25	1	Vancouver	–84.2	7.9	hair
H28	1	Burnaby	–82.4	8.6	hair
H30	1	Burnaby	–85.5	7.6	hair
T31	2	Orillia	–88.4	12.9	toenail
T32	2	Orillia	–88.1	6.7	toenail
H33	2	Barrie	–75.9	7.5	hair
T33	2	Barrie	–82.8	8.6	toenail
H34	2	Orillia	–81.6	6.4	hair
T34	2	Orillia	–91.2	8.4	toenail
H35	2	Barrie	–73.4	9.6	hair
T35	2	Barrie	–92.1	9.5	toenail
T36	2	Horseshoe Valley	–86.9	11.1	toenail
T37	2	Orillia	–82.8	6.2	toenail
H38	2	Barrie	–81.4	5.8	hair
T38	2	Barrie	–88.7	4.7	toenail
H39	2	Orillia	–81.9	8.0	hair
T39	2	Orillia	–86.9	6.2	toenail
H40	2	Washago	–81.0	11.0	hair
T40	2	Washago	–85.2	3.6	toenail
H41	2	Orillia	–81.2	11.0	hair
H42	2	Orillia	–74.5	12.5	hair
H43	2	Barrie	–80.9	11.1	hair
H44	2	Lafontaine	–69.8	11.1	hair
H45	2	Orillia	–73.9	10.9	hair
H46	2	Orillia	–74.9	10.2	hair

Sample ID	Site	Location	δ^2H	δ^{18}O	Tissue
H47	2	Barrie	–74.6	11.4	hair
H48	2	Orillia	–77.7	10.7	hair
H49	2	Orillia	–76.0	9.6	hair
H50	2	Orillia	–74.6	9.9	hair
H51	2	Gravenhurst	–78.3	8.4	hair
H52	2	Orillia		11.8	hair
H53	2	Orillia	–70.3	9.3	hair
H54	2	Barrie	–75.3	12.3	hair
H55	2	Orillia	–76.0	13.5	hair
H56	2	Orillia	–71.4	12.3	hair
H57	2	Midland	–76.2	12.7	hair
H58	2	Orillia	–76.5	14.6	hair
H59	2	Orillia	–71.2	10.7	hair
T61	3	Iqaluit	–85.6	2.7	toenail
H61	3	Iqaluit	–77.0	13.6	hair
T62	3	Iqaluit	–82.7	2.3	toenail
H62	3	Iqaluit	–79.9	14.1	hair
T63	3	Iqaluit	–84.8	4.3	toenail
H63	3	Iqaluit	–78.0	11.1	hair
H65	3	Iqaluit	–83.8	10.3	hair
T65	3	Iqaluit	–86.9	8.0	toenail
T67	3	Iqaluit	–97.6	5.7	toenail
H67	3	Iqaluit	–81.6	7.0	hair
T68	3	Iqaluit	–93.5	9.4	toenail
H68	3	Iqaluit	–79.9	8.7	hair
T91	4	Kings County	–89.2	6.9	toenail
H91	4	Kings County	–77.2	8.8	hair
T92	4	Wolfville	–83.3	10.5	toenail
H92	4	Wolfville	–78.4	9.2	hair
T94	4	Wolfville	–83.7	10.2	toenail

Sample ID	Site	Location	δ^2H	δ^{18}O	Tissue
H94	4	Wolfville	–75.0	7.7	hair
H95	4	Kentville	–69.8	7.2	hair
T95	4	Kentville	–92.4	10.8	toenail
T96	4	Kentville	–89.0	16.5	toenail
H96	4	Kentville	–65.7	18.5	hair
T97	4	Wolfville	–91.7	11.2	toenail
H97	4	Wolfville	–71.1	20.6	hair
T98	4	Wolfville	–80.5	9.8	toenail
H98	4	Wolfville	–67.6	17.3	hair
H99	4	Wolfville	–70.0	17.4	hair
T99	4	Wolfville	–87.6	11.1	toenail
H100	4	Berwick	–63.6	17.7	hair
T100	4	Berwick	–79.8	12.6	toenail
T101	4	Wolfville	–92.1	9.9	toenail
H101	4	Wolfville	–73.2	16.2	hair
H102	4	Wolfville	–66.3	10.6	hair
H103	4	Wolfville	–71.3	9.9	hair
H104	4	Wolfville	–72.1	10.9	hair
H105	4	Wolfville	–71.0	10.1	hair
H106	4	Wolfville	–67.0	11.8	hair
H107	4	Kentville	–64.8	7.9	hair
H108	4	Wolfville	–74.4	10.2	hair
H109	4	Wolfville	–71.4	9.3	hair
H110	4	Wolfville	–78.7	10.2	hair
H111	4	Wolfville	–73.4	9.8	hair
H112	4	Wolfville	–71.8	10.0	hair
H113	4	Wolfville	–76.6	9.9	hair
H114	4	Wolfville	–71.5	10.0	hair
H115	4	Wolfville	–77.3	11.7	hair
H116	4	Kentville	–80.1	13.0	hair

Sample ID	Site	Location	δ^2H	δ^{18}O	Tissue
H117	4	Kentville	–71.6	14.1	hair
H118	4	Kentville	–76.7	14.0	hair
H119	4	Wolfville	–81.6	9.6	hair

*an outlier as identified by measuring the Mahalanobis distance metric[60].

Table S2. Tukey HSD Multiple pairwise comparisons as a post–hoc test (ANOVA.R)

Tissue	Isotope	Site 1	Site 2	Mean difference	lower	Upper	p.adj
Hair	δ^2H	1	2	6.32	1.99	10.64	< 0.00*
	δ^2H	1	3	2.49	–4.30	9.27	0.77
	δ^2H	1	4	10.03	5.87	14.12	< 0.00*
	δ^2H	2	3	–3.83	–10.59	2.92	0.45
	δ^2H	2	4	3.71	–0.40	7.83	0.09
	δ^2H	3	4	7.55	0.89	14.21	< 0.05*
	δ^{18}O	1	2	2.48	0.18	4.79	< 0.05*
	δ^{18}O	1	3	2.84	–0.78	6.46	0.18
	δ^{18}O	1	4	3.97	1.74	6.19	< 0.00*
	δ^{18}O	2	3	0.36	–3.25	3.96	0.99
	δ^{18}O	2	4	1.48	–0.72	3.68	0.29
	δ^{18}O	3	4	1.13	–2.43	4.68	0.84
Toenails	δ^2H	1	2	9.28	3.10	15.46	< 0.00*
	δ^2H	1	3	8.07	0.82	15.32	< 0.05*
	δ^2H	1	4	9.66	3.48	15.83	< 0.00*
	δ^2H	2	3	–1.21	–8.80	6.37	0.97
	δ^2H	2	4	0.37	–6.19	6.94	0.99
	δ^2H	3	4	1.59	–5.99	9.17	0.94
	δ^{18}O	1	2	–0.16	–3.07	2.75	0.99
	δ^{18}O	1	3	–2.57	–5.98	0.84	0.20
	δ^{18}O	1	4	2.99	0.08	5.90	< 0.05*
	δ^{18}O	2	3	–2.41	–5.98	1.16	0.28
	δ^{18}O	2	4	3.15	0.06	6.24	< 0.05*
	δ^{18}O	3	4	5.56	1.99	9.13	< 0.00*

*p.adj significance at the $\alpha = 0.05$ level

Table S3. Decision tree models' performance evaluation summary.

Model	N	Accuracy	Accuracy 95% CI	Accuracy p value	Kappa stats	Site	Positive predictive value	Negative predictive value	Sensitivity	Specificity
Model 1	81	0.8131	(0.4134, 0.8898)	0.01119	0.5402	1	1.0000	0.9167	0.8000	1.0000
						2	1.0000	0.7143	0.3333	1.0000
						3	N/A	N/A	N/A	1.0000
						4	0.5000	1.0000	1.000	0.5455
Model 2	39	0.4286	(0.099, 0.8159)	0.6407	0.3	1	1.0000	0.6667	0.3333	1.0000
						2	N/A	NA	NA	0.5714
						3	N/A	0.7143	0.0000	1.0000
						4	0.6667	1.0000	1.000	0.8000
Model 3	35	0.7143	(0.4213, 0.9964)	0.1243	0.7586	1	1.0000	1.0000	1.0000	1.0000
						2	N/A	N/A	N/A	0.8571
						3	N/A	0.8571	0.0000	1.0000
						4	1.0000	1.0000	1.0000	1.0000
